# Ethical misconduct by registered physiotherapists in South Africa (2007–2013): A mixed methods approach

**DOI:** 10.4102/sajp.v71i1.248

**Published:** 2015-06-24

**Authors:** Willem A. Hoffmann, Nico Nortjé

**Affiliations:** 1 Department of Biomedical Sciences, Tshwane University of Technology, South Africa; 2 Department of Dietetics, University of the Western Cape, South Africa

## Abstract

**Background:**

The role of ethics in a medical context is to protect the interests of patients. Thus, it is critically important to understand the guilty verdicts related to professional standard breaches and ethics misconduct of physiotherapists.

**Aim:**

To analyse the case content and penalties of all guilty verdicts related to ethics misconduct against registered physiotherapists in South Africa.

**Methods:**

A mixed methods approach was followed consisting of epidemiological data analysis and qualitative content analysis. The data documents were formal annual lists (2007–2013) of guilty verdicts related to ethical misconduct. Quantitative data analysis focused on annual frequencies of guilty verdicts, transgression categories and the imposed penalties. Qualitative data analysis focused on content analysis of the case content for each guilty verdict.

**Results:**

Relatively few physiotherapists (0.05%) are annually found guilty of ethical misconduct. The two most frequent penalties were fines of R5000.00 and fines of R8000.00 – R10 000.00. The majority of transgressions involved fraudulent conduct (70.3%), followed by performance of procedures without patient consent (10.8%). Fraudulent conduct involved issuing misleading, inaccurate or false medical statements, and false or inaccurate medical aid scheme claims.

**Conclusion:**

Unethical conduct by physiotherapists in South Africa occurs rarely. The majority of penalties imposed on sanctioned physiotherapists were monetary penalties.

… [*T*]hrough professionalism and a set of principles and commitments, it is possible to improve the patient's health outcome and maximize their independence, creating relationships characterized by integrity, ethical practice, social justice and teamwork. (Novaes *et al*. 2014:165)

## Introduction

Ethics is a discipline of thought and study regarding the moral principles of human behaviour; it is about right and wrong in conduct, often in accordance with explicit or implicit rules or standards as accepted within a specific context (Kirby 2009). In a health context it focuses on protecting patients’ interests, freedoms and well-being (Verheyden 2012). As a result, the conduct of most practitioners in formally recognised health professions across the world is guided and regulated by ethical standards and codes of conduct to protect the public at large and patients specifically. These standards and codes of conduct define foundational ethical principles, describe professional obligations, aim to raise accountability, commitment and professional integrity, whilst also promoting adherence and compliance to those standards (Banks 1998; Burke *et al*. 2007; Oyeyemi 2011). Furthermore, the codes can empower practitioners to make informed and reasoned decisions in real-life contexts that pose ethical dilemmas and challenges (Burke *et al*. 2007; Oyeyemi 2011). However, the written codes in themselves do not ensure ethical practice or avert health professionals from engaging in various forms of unethical behaviour in professional-patient and professional practice contexts; ethical conduct is more than simple compliance to rules and regulations (Banks 1998). Inherent ethical values such as professionalism, honesty and integrity are vitally important in these contexts to guide and motivate each health practitioner's ethical conduct. It motivates health practitioners to maintain the highest possible clinical skill levels and to stay informed of the latest advances, technologies and insights through continuous professional development activities (Scherrer, Louw & Möller 2002). In addition, ethics education must form an integral and compulsory part of all health-related undergraduate, postgraduate and continuous professional development programmes (Health Professions Council of South Africa [HPCSA] 2006). Essentially, ethics education aims to raise students’ and practitioners’ ethical self-awareness, ethical issue recognition, ethical decision making and the application of ethical principles and guidelines (Carlin *et al*. 2011).

The physiotherapy profession is widely recognised as the field of health sciences that focuses on the assessment, treatment and prevention of human movement disorders. Generally, it aims to restore normal function or to minimise physical dysfunction, impairment and pain in persons of all age groups; the ultimate aim being to facilitate the highest possible level of independence in adults and children (South African Society of Physiotherapy 2014). In terms of a code of ethics for physiotherapists, the American Physical Therapy Association (APTA) subscribes to the following professional core values: accountability, altruism, compassion, excellence, integrity, professional duty and social responsibility (APTA 2012). These values are also important in the South African context. Ethical conduct of physiotherapists registered in South Africa is regulated by the Professional Board for Physiotherapy, Podiatry and Biokinetics of the Health Professions Council of South Africa (HPCSA 2008). The HPCSA has formal jurisdiction to investigate any complaint or allegation of unprofessional conduct by the public or other health care practitioners against registered physiotherapists; this includes the power to institute disciplinary proceedings (*Health Professions Act* [No 56 of 1974], paragraphs 3[n], 3[o] and 41[1]). An analysis of recent HPCSA guilty verdicts can provide important insights into duly investigated ethics misconduct by registered physiotherapists in the South African health care context. In addition, such an analysis can provide valuable information for continuous professional development ethics training programmes. As such, the objectives of this study were the following:

- To analyse the case content of all guilty verdicts related to professional standard breaches and ethics misconduct of HPCSA-registered physiotherapists in the period 2007–2013.
- To analyse the penalty content of all guilty verdicts related to professional standard breaches and ethics misconduct of HPCSA-registered physiotherapists during the same period.

## Methods

A mixed methods approach was followed in this study, specifically an explanatory sequential design that is characterised by the collection and analysis of quantitative data followed by the collection and analysis of qualitative data (Creswell 2013). The quantitative component of the study focused on the following annual frequency data (2007–2013) regarding the number of sanctioned practitioners:

- The physiotherapists who were found guilty of professional misconduct in terms of the ethical rules of conduct for HPCSA-registered health care practitioners (HPCSA 2008).
- The number of guilty verdict cases, that is all the specific cases brought against physiotherapists who were found by the HPCSA to be actual cases of professional misconduct transgressions in terms of the ethical rules of conduct for HPCSA-registered health care practitoners (HPCSA, 2008).
- The number of specific penalties imposed by the HPCSA on guilty physiotherapists in terms of the *Health Professions Act* (No 56 of 1974), paragraph 42(1).

Any acts or omissions by HPCSA-registered health practitioners to comply with the ethical rules of conduct for HPCSA-registered health care practitioners (HPCSA 2008) can be found guilty of professional misconduct. The various penalties that may be imposed on guilty practitoners include the following: a caution or reprimand; suspension from practicing for a specified period; removal of name from the register; prescribed fine; compulsory period of professional service; payment of the costs of the professional conduct proceedings or restitution. The execution of all penalties, or parts thereof, may be suspended for a period (*Health Professions Act* [No 56 of 1974], paragraphs 19[1][g], 19A[1][c], 42[2] and 43[1][b]). The qualitative research component of the study specifically used a historical research approach with archival material as the primary data source (Neuman 1997). The study archive was the annual lists (2007–2013) of the guilty verdict descriptions related to professional standard breaches and ethical misconduct against HPCSA-registered physiotherapists: the annual lists are accessible in the public domain on the HPCSA website (http://www.hpcsa.co.za/conduct_guilty_verdicts.php).

## Data analysis

Quantitative data analysis focused on the compilation of frequency tables for the following three variable combinations: annual frequency of sanctioned physiotherapists and guilty verdict cases; annual frequency of penalties imposed on sanctioned physiotherapists; and frequency of transgression categories linked to sanctioned physiotherapists. The qualitative data analysis focused on an investigation of the specific case content for each guilty verdict by means of qualitative content analysis (Neuman 1997). This involved a systematic coding and thematic description of each case. Initially each of the two researchers independently conducted the qualitative content analysis on selected annual guilty verdict documents, followed by several discussions until consensus was reached.

### Ethics exemption

Studies that exclusively focus on the analysis of publicly available documents are widely exempt from the requirement for ethics clearance from a registered research ethics committee (Department of Health 2004). In keeping with the ethics principle of anonymity, the sanctioned physiotherapists’ identifying information (specifically their names and HPCSA registration numbers) are not reported in this study although it is provided in the publicly available archival material.

## Results

### Frequency of guilty verdicts

The annual frequency of guilty verdicts against physiotherapists for the period 2007–2013 is indicated in Table 1. The results indicate that annually only a very small fraction of registered physiotherapists (average = 0.05%; range 0% – 0.10%) were found guilty of misconduct during the study period. The overall annual average number of guilty verdicts per sanctioned physiotherapist was 1.33 (range 1.00–1.75).

**TABLE 1:** Annual frequency of guilty verdicts against physiotherapists (2007–2013).

Year	Registered physiotherapists (*n*)	Guilty verdicts (*n*)	Sanctioned physiotherapists (*n*)	Sanctioned physiotherapists (%)	Guilty verdicts per sanctioned physiotherapist (Mean)
2007	5240	7	4	0.08	1.75
2008	5081	2	2	0.04	1
2009	5261	7	4	0.08	1.75
2010	5773	3	3	0.05	1
2011	5954	0	0	-	-
2012	6328	2	2	0.03	1
2013	6329	7	6	0.10	1.17
Mean	5709	4	3	0.05	1.33

### Penalties imposed to sanctioned physiotherapists

The annual number and overall relative percentage of the different penalties imposed on sanctioned physiotherapists in the period 2007–2013 is indicated in Table 2. The two most frequent penalties imposed on sanctioned physiotherapists were fines of R5000.00 (26%) and fines between R1000.00 and R3000.00 (15%). These were followed by fines between R8000.00 and R10 000.00, and cautions or caution-and-reprimands; each being imposed on 11% of cases. Combined these penalties constitute approximately 63% of all the penalties.

**TABLE 2:** Annual frequency of penalties imposed on sanctioned physiotherapists (2007–2013).

Penalty	2007†	2008†	2009†	2010†	2011†	2012†	2013†	% of all penalties
Caution or Caution-and-reprimand	1	1	-	-	-	-	1	11.11
Fine: R1000.00 to R3000.00	2	-	1	-	-	-	1	14.81
Fine: R5000.00	1	-	1	1	-	2	2	25.92
Fine: R8000.00 to R10 000.00	-	-	-	2	-	-	1	11.11
Suspension: 3 months	-	1	-	-	-	-	-	3.70
Suspension: 1 year	-	-	1	-	-	-	1	7.41
Suspension: 3 years	2	-	-	-	-	-	-	7.41
Erase from register	-	-	1	-	-	-	-	3.70
Attendance of ethics course	-	-	1	1	-	-	-	7.41
Attendance of practice management course	1	-	1	-	-	-	-	7.41

†, number.

The frequency of the different penalties imposed for the various transgression categories in the period 2007–2013 is indicated in Table 3. A wide variety of penalties were imposed for fraudulent conduct. Issuing false and/or inaccurate medical aid claims involving non-rendered services were given the following range of penalties: caution-and-reprimand; R3000.00 fine and 3 year suspended suspension; R5000.00 fine only; R8000.00 fine only; or 12 months suspended suspension. The latter penalty was imposed as the physiotherapist was also found guilty of issuing misleading, inaccurate and/or false medical statements. Other physiotherapists found guilty of issuing misleading, inaccurate and/or false medical statements were given a R5000.00 fine with the requirement to attend a course in ethics in one case and a 12 months suspended suspension with the requirement to attend a course in practice management in another case. A suspended suspension of 3 months was imposed for misrepresentation of professional status.

**TABLE 3:** Frequency of penalties imposed on transgression categories.

Penalty	Fraudulent conduct	Perform procedures / interventions without patient consent	Improper professional role conduct	Negligence / incompetence in evaluating, treating or caring for patients	Negligence regarding patient documents / records
Caution or Caution-and-reprimand	1	2	-	-	-
Fine: R1000.00 to R3000.00	3	-	-	-	1
Fine: R5000.00	4	1	1	1	-
Fine: R8000.00 to R10 000.00	2	2	-	-	-
Suspension: 3 months	1	-	-	-	-
Suspension: 1 year	1	-	-	-	-
Suspension: 3 years	2	-	-	-	-
Erase from register	1	-	1	1	-
Attendance of ethics course	-	1	1	-	-
Attendance of practice management course	1	1	-	-	-

A wide variety of penalties were also imposed in cases where procedures and interventions were performed without patient consent. It ranged from a caution-and-reprimand in one case to a R10 000.00 fine with the requirement to attend a course in ethics in another case where the physiotherapist failed to inform the patient of the risks and potential complications associated with a specific procedure. Other notable penalties involved fines of R5000.00 for improper professional role conduct (i.e. a romantic relationship with a married patient who is being treated at the same time) and negligence in caring for patients by treating a dog at a practice which exposed patients to unjustified danger. Arguably the most serious penalty, namely removal from the HPCSA register, was imposed in only one case during the study period; the physiotherapist was found guilty of various transgressions, namely providing inappropriate/redundant treatment to patients, negligent record keeping, consulting patients without medical practitioner referrals, charging patients for non-rendered services and overcharging patients.

### Transgressions committed by sanctioned physiotherapists: Quantitative analysis

The frequency of transgression categories linked to the guilty verdicts against physiotherapists across the total study period (2007–2013) is indicated in Table 4. The vast majority of transgressions involve fraudulent conduct (70.3%), followed by the performance of procedures/interventions without patient consent (10.8%). The rest of the transgressions combined (18.9%) occurred in three transgression categories, namely improper professional role conduct, negligence in evaluating, treating or caring for patients, and negligence regarding patient documents or records.

**TABLE 4:** Frequency of transgression categories linked to transgression cases.

Transgression category	Number of transgression cases *	% of all transgression cases
Fraudulent conduct	26	70.30
Perform procedures/interventions without patient consent	4	10.80
Improper professional role conduct	3	8.10
Negligence or incompetence in evaluating, treating or caring for patients	2	5.40
Negligence regarding patient documents or records	2	5.40

Note: The number of transgression cases outnumber the number of sanctioned physiotherapists and/or the number of penalties because of the fact that some physiotherapists were found guilty of more than one transgression case at a specific ethics misconduct hearing.

### Transgressions committed by sanctioned physiotherapists: Qualitative analysis

The qualitative analysis of specific ethical misconduct by sanctioned physiotherapists within each transgression category is indicated in Table 5. The issuing of misleading, inaccurate and/or false medical statements together with false and/or inaccurate medical aid claims involving non-rendered services were found to be the most important specific ethics misconduct linked to fraudulent conduct. Improper professional role conduct involved cases where patients were consulted without due medical practitioner referrals, as well as the utterance of derogatory/abusive remarks towards patients and being in a romantic relationship with a married patient. Negligence emerged in two qualitative transgression categories. In the first category, negligence/incompetence in evaluating, treating and caring for patients involved over-servicing patients by performing redundant procedures. In the second category, negligence regarding patient documents and records, involved the failure to keep proper patient records and the failure to timeously submit account statements to the relevant medical aid scheme.

**TABLE 5:** Specific ethical misconduct by sanctioned physiotherapists within each transgression category.

Transgression category	Specific ethical misconduct (in descending order of frequency)
Fraudulent conduct	• Issuing misleading, inaccurate and/or false medical statements • False and/or inaccurate medical aid claims involving non-rendered services • Misrepresentation of professional status – claiming to be a ‘doctor’ when not registered as such
Perform procedures and interventions without patient consent	• Failure to obtain patient consent to perform procedures and interventions
Improper professional role conduct	• Consult patient without medical practitioner referral • Express verbal derogatory or abusive remarks towards patient • Romantic relationship with married patient whilst person is also being treated as a patient
Negligence or incompetence in evaluating, treating and caring for patients	• Over-service patient by performing redundant procedures • Treating an animal (dog) at practice, thereby exposing patients to unjustified danger
Negligence regarding patient documents and records	• Failure to keep proper patient records • Failure to timeously submit statement of account to medical aid scheme, resulting in financial prejudice to patient

## Discussion

The annual frequency of sanctioned physiotherapists in the period 2007–2013 indicates that on average only 0.05% physiotherapists are annually found guilty of ethical transgressions. The total of 37 transgressions across the study period (7 years) for an annual average of 5709 registered physiotherapists in South Africa is slightly higher than the total of five serious ethical transgressions for the period 2006–2013 (8 years) for approximately 4200 registered practitioners during the same period in New Zealand (New Zealand Health Practitioner Disciplinary Tribunal [NZHPDT] 2014). However, the South African figures compare favourably with the annual frequency of 0.09% sanctioned annual disciplinary enquiries against HPCSA-registered psychologists during 2007–2013 (Nortjé & Hoffmann 2015) and the average annual frequency of 0.17% sanctioned medical practitioners in South Africa during the same period (Hoffmann & Nortjé in press). The overall mean number of guilty verdicts per sanctioned physiotherapist was slightly above one verdict (1.33), suggesting that sanctioned practitioners generally only engage in once-off acts of misconduct. One can at this stage merely speculate on the reason(s) for the lower frequency of misconduct amongst physiotherapists in South Africa when compared to other health care practitioners, but it might be in part as result of any of the following:

- The higher potential for physical and physiological risks associated with medical interventions, pharmacological drugs and surgery by medical practitioners.
- The higher potential for fraudulent medical aid claims involving substantial amounts by medical practitioners.
- Effective ethics training for physiotherapists during undergraduate education and/or continuous professional development programmes.

A frequency analysis of the penalties imposed on sanctioned physiotherapists across the study period (Table 2) indicates that the HPCSA mostly opted to impose financial penalties (53.6%), whilst suspended suspension penalties from as short as 3 months up to a maximum of 3 years were imposed in 17.9% of the cases. Across the study period of 7 years (2007–2013) only one physiotherapist was removed from the HPCSA register because of multiple transgressions involving *inter alia* rendering inappropriate treatment to patients, negligent recordkeeping and fraudulently charging patients for non-rendered services (Table 3). Deregistration provides the ultimate protection for society against unscrupulous health care practitioners whilst also making an unambiguous statement about expected professional standards (Godbold 2008). In some of the cases involving issuing misleading, inaccurate and/or false statements, or failure to obtain patient consent to perform procedures or interventions, or making derogatory /abusive remarks towards a patient, the sanctioned physiotherapists were ordered to attend a practice management course (7.4%, *n* = 2) or to attend a medical ethics and law course (7.4%, *n* = 2) (Table 3). This is substantially higher than the 1.10% (*n* = 5) of sanctioned medical practitioners in South Africa who were required to attend a medical ethics course in the period 2007–2013 (Hoffmann & Nortjé in press); however, the low numbers of actual cases for physiotherapists (*n* = 2) and medical practitioners (*n* = 5) respectively in this regard indicate that any statistical interpretation should be approached with caution to avoid undue bias. The various penalties imposed to physiotherapists by the HPCSA indicate that the following transgressions are viewed as serious ethical misconduct: issuing misleading, inaccurate and/or false medical statements; false and/or inaccurate medical aid claims involving non-rendered services; over-servicing patients by performing redundant procedures; and failure to obtain patient consent to perform procedures and interventions, especially when it involves risks and potential complications.

### Fraudulent conduct

Fraudulent conduct (70.3%) was by far the most common transgression by sanctioned physiotherapists in the study period (Table 4). Similar to the findings of a recent study by Ogubanjo and Knapp van Bogaert (2014) on health care fraud in South Africa, the current study found the following specific fraudulent conduct activities: issuing misleading, inaccurate or false medical statements, and submitting false or inaccurate medical aid claims involving non-rendered services. In addition, over-servicing patients in terms of negligently performing redundant procedures or failure to timeously submit patient account statements to a medical aid scheme can also result in undue or prejudiced medical aid claims. Such fraudulent conduct constitutes a contravention of the HPCSA's Ethical and Professional Rules, specifically section 7(5) that deals with fees and commission (HPCSA 2008). The relative frequency of fraudulent-related transgressions in relation to all ethical transgressions for physiotherapists in this study is higher than what was found for other health practitioners in South Africa during the same period (2007–2013): dentists (55%) (Nortjé & Hoffmann 2014), medical practitioners (48.4%) (Hoffmann & Nortjé in press) and psychologists (21%) (Nortjé & Hoffmann 2015). These findings support the opinion of Ogubanjo and Knapp van Bogaert (2014) that health care fraud is a serious ethical issue for many health care practitioners in South Africa, especially as this type of fraudulent personal gain behaviour almost always results in direct or indirect prejudice to the patient and medical aid scheme. It invariably also involves issues around trust, dishonesty and abuse of professional position (Godbold 2008). The HPCSA is justifiably taking a firm stand on fraudulent conduct by imposing fines of up to R10 000.00, a 3 year suspension from practicing (suspended) or even removal from the register. In New Zealand similar penalties (6 months suspension and cancellation of registration respectively) were imposed by the NZHPDT in the period 2006–2014 to two physiotherapists found guilty of fraud related to false/inaccurate medical claims involving non-rendered services and forgery (Godbold 2008; NZHPDT 2014).

### Consent

The HPCSA's ethical rules of conduct for practitioners registered under the *Health Professions Act* (No. 56 of 1974) clearly indicates that one of the main practitioner responsibilities is to obtain prior, free and informed patient consent for any therapeutic intervention and procedure (Paragraph 27A[g], HPCSA 2008). This principle is universally recognised (United Nations Educational Scientific and Cultural Organisation [UNESCO] 2006) and forms the foundation of mutual trust and respect in patient-physiotherapist interactions. Furthermore, it empowers patients to freely explore a variety of therapeutic options and therapeutic modalities (Paragraph 27A[d], HPCSA 2008). In the light of the fundamental nature of consent in the patient-therapist relationship, it is rather disconcerting that failure to obtain patient consent to perform procedures and/or interventions was the second most frequent transgression category (10.8%) amongst sanctioned physiotherapists in the period 2007–2013 (Table 4).

### Improper professional role conduct

The *Health Professions Act* (No.56 of 1974) in South Africa defines *unprofessional conduct* as:

… [*I*]mproper or disgraceful or dishonourable or unworthy conduct or conduct which, when regard is had to the profession of a person who is registered in terms of this Act, is improper or disgraceful or dishonourable or unworthy. (n.p.)

It is closely associated with two of the main responsibilities of HPCSA-registered practitioners (HPCSA 2008), namely to maintain the highest standards of personal conduct and integrity (Paragraph 27A[c]) and to respect patient privacy, choices and dignity (Paragraph 27A[b]); see also UNESCO (2006), articles 2(c) and 3(1). In light of these principles improper professional role conduct in expressing derogatory or abusive remarks towards a patient constitutes a direct form of disrespect for patient dignity. Another form of improper professional role conduct involves being involved in a romantic relationship with a married patient whilst the person is also being treated as a patient. Such relationships could reasonably be argued to impair the physiotherapist's professional judgement to act in the best interest of the patients, as well as posing a risk of patient exploitation. In New Zealand the NZHPDT, similar to the situation in South Africa, only found three physiotherapists guilty of unprofessional conduct, specifically referred to as *inappropriate behaviour*, during the period 2006–2013. However, the New Zealand cases involved inappropriate touching/exposure of female patients’ breasts (all three cases) together with inappropriate romantic advances/comments in one case. The penalties in these cases ranged from a fine, compulsory mentoring programme attendance, compulsory ethics course attendance and/or cancellation of registration (NZHPDT 2014).

## Conclusion and recommendations

The most significant contributions of this study are the results regarding the frequency of transgression categories, the frequency of penalties imposed on the various transgression categories, as well as the specific ethical misconduct by physiotherapists within each transgression category. The most encouraging finding is that ethical transgressions by physiotherapists in South Africa occur rarely; only 0.05% of all HPCSA-registered physiotherapists during the period 2007–2013 were found guilty of unethical professional conduct.

Another significant finding is that more than half (52%) of the penalties imposed on sanctioned physiotherapists were monetary penalties between R1000.00 and R10 000.00, followed by suspended suspensions between 3 months and 3 years (18.5%). However, in the study context of *ethical misconduct* transgressions it was somewhat surprising that only 7.4% of the guilty verdicts ordered the physiotherapist to specifically attend an *ethics* course, whilst another 7.4% of the guilty verdicts ordered attendance of a *practice management* course. This raises some concerns regarding the justification and effectiveness of the imposed penalties to rehabilitate offending practitioners (Godbold 2008), especially as the highest monetary penalties in our study were linked to the most frequent transgressions throughout the study period, namely fraudulent conduct. This suggests that the current monetary penalties are possibly not high enough to be effective deterrents; penalties with more serious professional consequences should be considered, such as actual suspensions which are not suspended, and erasure from the register for a specified period with strict training requirements before re-instatement on the register. However, an analysis of physiotherapist income levels and the potential impact of monetary fines on their expendable income falls outside the scope of this study and should be pursued in subsequent studies. The last significant finding is that fraudulent conduct was by far the most frequent transgression category, specifically issuing misleading, inaccurate and/or false medical statements, as well as submitting false and/or inaccurate medical aid claims involving non-rendered services.

In conclusion, the following three recommendations are suggested to further enhance ethical conduct amongst physiotherapists in South Africa:

- The relationship between ethics education and ethical behaviour is complex. As such, systematic and comprehensive ethics training must form an integral and compulsory part of all health-related undergraduate, postgraduate and continuous professional development programmes (HPCSA 2006). Although ethics training modules already form part of physiotherapy programmes, its long-term effectiveness is *inter alia* determined by the skills of students and practitioners to apply ethics principles to their specific practices in a critical, logical, analytic and informed way (Banks 1998; Oyeyemi 2011). Often students are taught the principles without engaging them in a Socratic debate where their own moral development is investigated. Changing the focus of ethics education to engage in a higher moral awareness rather than an application of principles is critical to develop a skill of reflection, which is necessary once the students become registered practitioners.
- Professional ethics awareness should involve more than mere awareness and/or adherence to HPCSA and/or the South African Society of Physiotherapy codes of conduct. Some practitioners may be of the opinion that mere compliance to the rules and guidelines of these codes constitute ethical conduct, whilst in actual fact the intention of codes of conduct is to engage practitioners in in-depth ethical reflection, interpretation, critical thinking, debate and decision-making skills to deal with a wide variety of ethical dilemmas in health care contexts (Banks 1998; Novaes *et al*. 2014). The codes should also assist physiotherapists to bridge the divide between abstract theoretical ethics principles and constructs contained in medico-legal documents and on-the-ground-real-life ethical challenges (Burke *et al*. 2007). The various continuous professional development activities for physiotherapists in South Africa can play an increasingly integral role in this endeavour.
- The primary purpose of penalties for ethical transgressions is to protect the public and health care professional standards, specifically in terms of providing a deterrent to others. However, punishment per se should not be the main aim of sanctions (Godbold 2008). As such, the HPCSA should reconsider the predominantly punitive nature of sanctions imposed to physiotherapists found guilty of ethics transgressions. Fines and suspended suspensions are penalties that in themselves do not rehabilitate sanctioned physiotherapists or even facilitate professional and ethical conduct (Godbold 2008). In order to enhance the rehabilitation of these practitioners and to facilitate professional development the authors strongly recommend that all sanctioned physiotherapists be ordered to complete a duly accredited medical ethics course within a specified period of time. Such a corrective measure will closely align with the HPCSA's view (2008:3) that ‘… to be a good health care practitioner, requires a life-long commitment to sound *professional and ethical practices* …’ (emphasis added).
